# Heart Failure in the Molecular Era: Redefining Our Understanding of Disease Mechanisms and Perspectives

**DOI:** 10.3390/biomedicines14020486

**Published:** 2026-02-23

**Authors:** Manuel Mallol-Simmonds, Alfredo Parra-Lucares, Ivan Canete, Cristian Avila, Josseline Pena-Silva, Sergio Bustamante

**Affiliations:** Cardiovascular Investigation Department, University of Chile’s Clinical Hospital, Santiago 8380456, Chile; manuel.mallol@ug.uchile.cl (M.M.-S.);

**Keywords:** heart failure, renin–angiotensin system, microbiota, genomics, stem cell therapy, genetic therapy, personalized medicine, oxidative stress

## Abstract

Heart failure (HF) is a global health challenge characterized by the heart’s inability to satisfy metabolic demands, driven by renin–angiotensin–aldosterone system (RAAS) overactivation, a neurohormonal imbalance, and emerging mechanisms like the gut–heart axis and mitochondrial dysfunction. Affecting over 6 million adults in the US alone, HF incurs a 5-year mortality rate of 50% and escalating costs projected to double by 2030. This review examines HF’s molecular paradigms, integrating established pathways with advances in omics, stem cell therapy, genetic modification, and personalized medicine. The RAAS blockade remains central, yet its efficacy is limited in HF with preserved ejection fraction (HFpEF). Stem cell therapies (mesenchymal and induced pluripotent stem cells) show regenerative potential but face poor retention (<10% survival at 30 days). CRISPR/Cas9 offers precision, though off-target effects persist. The gut microbiome, via trimethylamine N-oxide, exacerbates inflammation, while omics technologies promise biomarkers for tailored treatments. Challenges include translating these innovations into practice, particularly for HFpEF. Future directions involve novel HFpEF therapies, enhanced stem cell delivery, precise genetic tools, and microbiome interventions, supported with artificial intelligence. By 2030, these advances could shift HF management toward regeneration, contingent on overcoming translational barriers through global collaboration.

## 1. Introduction

Heart failure (HF) is a complex clinical syndrome defined by the heart’s inability to deliver adequate cardiac output to meet the body’s metabolic demands, often necessitating elevated ventricular filling pressures to maintain circulation [1]. This condition imposes a profound global health burden, with significant epidemiological and economic implications. In the United States, HF affects approximately 6.2 million adults (2013–2016), with an annual incidence of 915,000 new cases and a projected 46% increase in prevalence by 2030, driven by an aging population and persistent risk factors such as systemic arterial hypertension and type 2 diabetes mellitus [2]. In Europe, prevalence ranges from 1 to 2% in adults, escalating to over 10% among those aged 70 and older, with elevated rates in Germany and the United Kingdom due to ischemic heart disease [3]. Japan reports a prevalence of 1–1.2%, while Brazil’s is 1–2%, reflecting a growing adoption of Western cardiovascular risk profiles [4,5]. The morbidity of HF is characterized by recurrent episodes of acute decompensation, leading to frequent hospitalizations, and a 5-year mortality rate approaching 50%, comparable to certain aggressive malignancies [6]. Economically, HF-related costs in the US surpassed $30.7 billion in 2012 and are projected to double by 2030, while Europe faces an annual burden of approximately €29 billion, underscoring the urgent need for optimized therapeutic strategies [7,8].

The pathophysiology of HF involves myocardial contractile dysfunction, oxidative stress, and adverse ventricular remodeling, primarily mediated by an overactivation of the renin–angiotensin–aldosterone system (RAAS) and the release of proinflammatory cytokines, such as tumor necrosis factor-alpha (TNF-α) [9]. Recent research has elucidated novel mechanisms, including the epigenetic regulation of cardiomyocyte gene expression, the gut–heart axis mediated by the microbiome, and mitochondrial dysfunction, all of which are under active investigation as potential therapeutic targets [10]. This review aims to provide a comprehensive overview of HF’s molecular paradigms, seamlessly integrating established knowledge with cutting-edge research to explore their applicability across diverse global contexts. By bridging traditional and emerging perspectives, we seek to redefine the understanding of HF and highlight pathways toward transformative clinical interventions.

This review was conducted as a narrative synthesis of current literature, aiming to integrate classical and emerging molecular mechanisms underlying HF. Relevant articles were identified through targeted searches in databases such as PubMed, Scopus, and Web of Science, among others, using combinations of keywords and MeSH terms including “heart failure,” “RAAS,” “gut microbiota,” “omics,” “stem cell therapy,” “CRISPR,” and “personalized medicine.” Preference was given to peer-reviewed studies published in the last 10 years, with seminal older works included where conceptually necessary. Studies were selected based on their relevance to the molecular pathophysiology of, and therapeutic innovation in, HF, with particular emphasis on translational potential and clinical applicability.

## 2. Classic Metabolic Pathways in Heart Failure

The cornerstone and starting point of this review is the overactivation of the RAAS, a neurohormonal system pivotal to cardiovascular homeostasis [11]. In the short term, RAAS activation compensates for reduced cardiac output by enhancing myocardial contractility and promoting fluid retention, thereby maintaining systemic perfusion. However, chronic overactivation induces deleterious effects, including myocardial hypertrophy, fibrosis, and systemic vasoconstriction, which accelerate HF progression. This sustained neurohormonal dysregulation, first comprehensively described by Packer in 1992, remains a primary driver of HF, positioning pharmacological RAAS blockade—through angiotensin-converting enzyme inhibitors (ACEIs), angiotensin II receptor blockers (ARBs), and mineralocorticoid receptor antagonists (MRA)—as a foundational treatment strategy [12].

Reduced cardiac output in HF triggers a cascade of compensatory responses detected via peripheral arterial baroreceptors, which signal an underfilled vascular state [11]. These responses include increased heart rate and myocardial contractility via sympathetic nervous system (SNS) activation, sodium and water retention through RAAS, and peripheral vasoconstriction to sustain blood pressure. In heart failure with reduced ejection fraction (HFrEF), these mechanisms initially preserve cardiovascular stability but become maladaptive over time, contributing to disease progression. Under normal conditions, counter-regulatory systems, such as parasympathetic tone and natriuretic peptides, mitigate SNS and RAAS activity. In HF, however, diminished parasympathetic tone and increased resistance to natriuretic peptides amplify neurohormonal activation, exacerbating cardiac strain. Originally termed “neurohormones” due to their neuroendocrine origins, these molecules are now recognized to function through paracrine and autocrine pathways, broadening their role in HF pathophysiology.

### 2.1. Neurohormonal Activation

Circulating volume is tightly regulated by baroreceptors in the aortic arch and carotid sinus (sensing high pressure) and pulmonary mechanoreceptors (detecting low pressure), which relay inhibitory signals to the central nervous system (CNS) to modulate SNS outflow [13]. In HF, reduced cardiac output diminishes baroreceptor activity, leading to heightened SNS activation, which increases heart rates, enhances myocardial contractility, and induces peripheral vasoconstriction to redistribute blood flow. Additionally, neurogenic signaling disrupts the balance between sympathetic and parasympathetic systems, with HF patients exhibiting increased chemosensitivity to hypoxia and hypercapnia, as well as exaggerated ergoreceptor reflexes triggered by metabolic byproducts of muscle activity [13,14]. These alterations impair functional capacity, reduce exercise tolerance (evidenced by decreased peak oxygen consumption, VO_2_), and correlate with disease severity and poorer survival prognosis. Elevated circulating neurohormone levels, sufficient to induce ventricular dysfunction and remodeling, underscore the therapeutic efficacy of SNS and RAAS inhibitors, which significantly improve clinical outcomes by attenuating these deleterious effects.

SNS overactivation, a hallmark of early HF, elevates circulating norepinephrine levels, amplifying adrenergic signaling through sustained release and reduced reuptake at nerve terminals [15]. This chronic stimulation promotes cardiomyocyte apoptosis, myocardial fibrosis, and endothelial dysfunction, exerting long-term detrimental effects on the heart, kidneys, and peripheral vasculature. Concurrently, RAAS activation amplifies these effects by increasing angiotensin II and aldosterone levels, further driving vasoconstriction and fluid overload, which contribute to left ventricular hypertrophy and dilatation, ultimately worsening HF progression [11,15].

### 2.2. Renal Function

**Figure 1:**
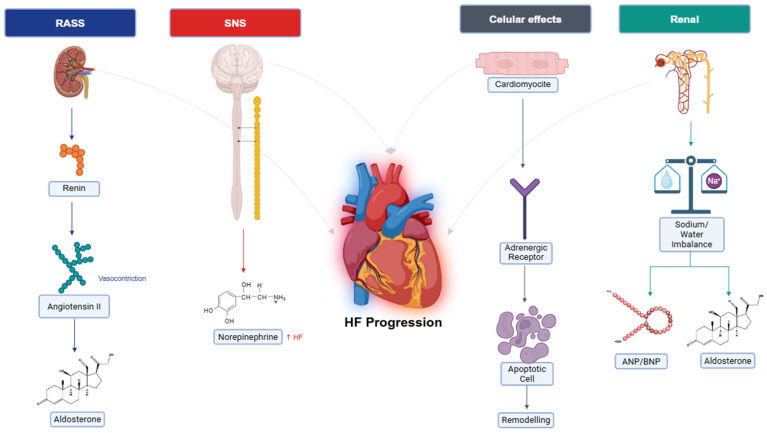
Simplified view of classical pathways in heart failure. Pathways are shown separately for clarity, but they interact extensively (not shown here).

Renal sodium and water retention, resulting in peripheral and pulmonary edema, is a central feature of HF, orchestrated by SNS and RAAS activation, rather than intrinsic renal pathology [16]. Sympathetic stimulation constricts the renal afferent arteriole, reducing glomerular blood flow and triggering renin release from the juxtaglomerular apparatus, an effect enhanced by β-adrenergic receptor activation. Renin catalyzes the conversion of hepatic angiotensinogen to angiotensin I, which angiotensin-converting enzyme (ACE) transforms into angiotensin II, a potent vasoconstrictor that binds to type 1 receptors (AT1) in the adrenal glomerulosa, stimulating aldosterone production. Angiotensin II also promotes proximal tubule sodium reabsorption and triggers vasopressin release from the hypothalamus, increasing water retention through non-osmotic pathways that override plasma osmolarity regulation, leading to hyponatremia [17]. Aldosterone, acting on distal nephrons, further enhances sodium retention, with levels rising 30–40% in HF patients despite ACEI or ARB use, a phenomenon known as “aldosterone escape” [17]. A schematic representation of the main pathways discussed above is shown in Figure 1. Counter-regulatory mechanisms involve natriuretic peptides, secreted in response to atrial and myocardial stretch, which promote cyclic guanosine monophosphate (cGMP)-mediated vasodilation and natriuresis. However, peripheral resistance to these peptides in HF, compounded by neprilysin-mediated degradation, limits their efficacy. Additional RAAS derivatives, such as angiotensin III (which stimulates aldosterone) and angiotensin 1–7 (which counteract ventricular remodeling), modulate this axis, though their therapeutic potential remains underexplored. These intricate renal and systemic interactions highlight the critical role of neurohormonal pathways in HF and inform targeted interventions, such as neprilysin inhibitors (e.g., sacubitril) combined with RAAS blockers, which enhance natriuretic peptide activity while suppressing deleterious RAAS effects.

## 3. Microbiome in Heart Failure: The Gut–Heart Axis

The systemic inflammation characteristic of HF, driven by RAAS overactivation and proinflammatory cytokines, is significantly amplified by the gut–heart axis, in which microbial dysbiosis plays a pivotal role in disease progression [18]. The gut microbiome, a diverse ecosystem of bacteria, viruses, archaea, bacteriophages, protozoa, and fungi, supports critical physiological functions, including metabolism, nutrient absorption, and immune system development [18,19]. Its composition exhibits considerable interindividual variability, shaped by environmental factors such as diet, medications, and lifestyle, and is characterized through metrics like alpha diversity (taxonomic richness and evenness within a sample), beta diversity (variability across samples), and gamma diversity (total richness across a habitat) [20]. Disruptions in microbiome composition, both qualitative and quantitative, are implicated in the pathogenesis of prevalent conditions, including obesity, metabolic syndrome, chronic kidney disease, hepatic steatosis, and cardiovascular disorders such as acute myocarditis, coronary artery disease, atrial fibrillation, and HF [21,22].

### 3.1. Gut Microbiome and Heart–Gut Axis

In HF, cardiac dysfunction compromises intestinal barrier function, leading to ischemia and edema of the gut wall, which increases permeability and allows the translocation of microorganisms and their metabolic byproducts, such as lipopolysaccharides, into the portal and systemic circulation [23]. This process elicits immune and inflammatory responses, establishing a bidirectional feedback loop that exacerbates cardiac dysfunction, as proposed by the “gut hypothesis” of HF [18]. Dysbiosis often precedes clinical HF, influenced by factors like Western diets rich in processed foods, sedentary lifestyles, circadian rhythm disruptions, and aging [24]. Notably, microbiome profiles in HF patients resemble those in dysmetabolic individuals before cardiovascular disease onset, suggesting a preclinical role in disease progression [24]. In advanced HF, pathogenic genera such as Shigella, Campylobacter, and Salmonella proliferate, contributing to persistent T-cell activation and increased susceptibility to Clostridioides infections, particularly in hospitalized patients receiving antibiotics [18]. Concurrently, anti-inflammatory taxa, including Coriobacteriaceae, Erysipelotrichaceae, Ruminococcaceae, Faecalibacterium, Eubacterium, Dorea, and Bifidobacterium, decline, reducing the production of butyrate, a key anti-inflammatory metabolite that modulates cytokine cascades and regulatory T-cell activity [23].

### 3.2. Disorders of Intestinal Metabolism in HF

HF-induced intestinal hypoxia and visceral venous congestion, particularly in right-sided HF, impair blood flow to epithelial cells, triggering cellular hypoxia, anaerobic metabolism, and the overexpression of the sodium/hydrogen exchanger 3, which increases sodium transport and lowers luminal pH [25]. Given that up to 40% of total blood volume resides in the gastrointestinal tract, these metabolic shifts have profound clinical implications [26]. Functional dysbiosis disrupts nutrient digestion, vitamin synthesis, and mucosal immunity, with a notable reduction in Bacteroides and Bifidobacteria and an increase in Firmicutes and Proteobacteria [26]. Fecal samples from chronic HF patients reveal higher concentrations of enteric pathogens (e.g., Salmonella, Shigella, and Campylobacter), correlating with disease severity and systemic inflammation and further straining cardiac function [18].

### 3.3. Trimethylamine N-Oxide 

**Figure 2:**
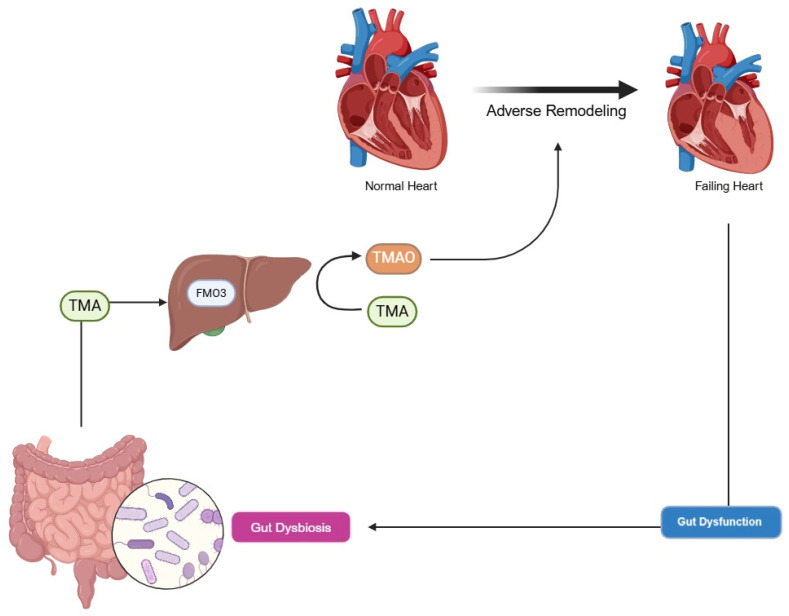
Gut–heart axis and its perpetuation. Intestinal dysbiosis, further sustained by dysfunction secondary to heart failure (HF), leads to an increase in TMA, which is converted into TMAO via hepatic FMO3. TMAO, along with other external agents, enhances adverse cardiac remodeling, perpetuating the cycle. Conceptual adaptation based on Ref. [27].

Trimethylamine N-oxide (TMAO), a metabolite produced by gut microbiota from dietary precursors like choline, betaine, L-carnitine, and phosphatidylcholine (found in seafood, dairy, eggs, meat, and organ meats), is increasingly recognized as a cardiovascular risk factor, often termed the “missing link” between Western diets and HF [19]. Gut bacteria, particularly Firmicutes and Proteobacteria (e.g., Providencia rettgeri and Clostridioides sporogenes), hydrolyze these nutrients via enzymes like choline-TMA lyase (cutC/D) and carnitine monooxygenase (cntA/B), producing trimethylamine (TMA). TMA is absorbed and oxidized to TMAO by hepatic flavin-containing monooxygenase (FMO3), with mutations in FMO3 causing trimethylaminuria due to TMA accumulatio. In HF, elevated TMAO levels correlate with increased inflammation, oxidative stress, and adverse ventricular remodeling, amplifying disease progression, as seen in Figure 2.

### 3.4. Strategies for TMAO Reduction 

Reducing TMAO levels holds a significant therapeutic promise for disrupting the gut–heart axis and mitigating HF progression. Several evidence-based strategies target TMAO production by modulating gut microbiota, inhibiting its synthesis, or altering dietary precursors, each offering distinct clinical potential. Some of these strategies are the following:

a. Dietary interventions: Diets rich in TMA precursors, such as red meat, eggs, and seafood, fuel TMAO production [19]. In contrast, Mediterranean or plant-based diets, low in these precursors and high in fiber, promote the growth of anti-inflammatory taxa (e.g., Bifidobacterium and Faecalibacterium) while reducing TMA-producing bacteria [27]. A 2020 randomized controlled trial demonstrated that a Mediterranean diet reduced plasma TMAO levels by 30% over 12 weeks in patients with cardiovascular risk factors, correlating with decreased inflammatory markers, including C-reactive protein and interleukin-6 [28]. Fiber-rich diets also enhance the production of short-chain fatty acids (SCFAs), such as butyrate, which mitigate gut dysbiosis and systemic inflammation, offering a practical and accessible approach for HF patients [29].

b. Probiotics and prebiotics: Probiotics, such as Lactobacillus and Bifidobacterium strains, and prebiotics (e.g., inulin and fructooligosaccharides) modulate gut microbiota to suppress TMA-producing bacteria. A 2021 clinical trial in patients with chronic HF showed that Lactobacillus rhamnosus supplementation (10^9^ CFU/day) for 8 weeks reduced TMAO levels by 25% and improved left ventricular ejection fraction (LVEF) by 4.2%, attributed to enhanced gut barrier function and reduced lipopolysaccharide translocation [30]. Prebiotics similarly foster SCFA production, with a meta-analysis reporting a 15–20% reduction in TMAO levels in cardiometabolic diseases, highlighting their potential as adjunctive therapies [31].

c. Pharmacological inhibition: Small-molecule inhibitors targeting microbial enzymes like choline-TMA lyase (cutC/D) and carnitine monooxygenase (cntA/B) prevent TMA formation, offering a targeted approach to TMAO reduction. 3,3-Dimethyl-1-butanol (DMB), a structural analog of choline, inhibits TMA production by gut bacteria. In a 2022 murine HF model, DMB administration reduced plasma TMAO by 40%, decreased myocardial fibrosis by 25% and improved LVEF by 10% at 8 weeks (*p* < 0.01), demonstrating robust cardioprotective effects [32]. Clinical trials are underway to evaluate DMB’s safety and efficacy in humans, with phase I studies reporting no significant adverse effects, suggesting a promising future for this approach [22]. However, antibiotics like rifaximin, which target Gram-negative bacteria, only transiently reduce TMAO and are limited by risks of microbial resistance and dysbiosis, making them less viable for long-term use [33].

d. Fecal microbiota transplantation (FMT): FMT aims to restore gut microbiota diversity by transferring healthy donor microbiota to HF patients. A 2023 pilot study reported a 35% reduction in TMAO levels at 12 weeks post-FMT, accompanied by improved exercise capacity (6 min walk test, +45 m, *p* = 0.03) and reduced brain natriuretic peptide levels, indicating potential cardiovascular benefits [34]. Despite these encouraging results, FMT’s scalability is constrained by donor variability, regulatory challenges, and uncertainties regarding long-term efficacy, necessitating further research to establish its clinical viability.

Despite these promising strategies, TMAO reduction faces several challenges. Interindividual variability in gut microbiota composition affects intervention efficacy, requiring personalized approaches to optimize outcomes. Long-term adherence to dietary or pharmacological regimens poses a practical barrier, particularly for patients with complex HF management needs. Safety concerns, such as off-target effects of enzyme inhibitors or rare infections associated with FMT, warrant careful investigation. Looking ahead, large-scale clinical trials are essential to confirm the impact of TMAO reduction on hard clinical endpoints, such as mortality and hospitalization rates, particularly in HFpEF, where therapeutic options remain limited [35]. Personalized interventions, leveraging microbiome sequencing to tailor dietary or probiotic regimens, and AI-driven models to predict TMAO responses, are anticipated to enhance efficacy by 2030 [36]. Furthermore, combining TMAO-targeted therapies with established treatments, such as RAAS inhibitors or SGLT2i, could synergistically address HF’s multifactorial pathophysiology, meriting exploration in phase II/III trials to establish their role in clinical practice.

## 4. The Evolution of Omics in Heart Failure Research

**Figure 3:**
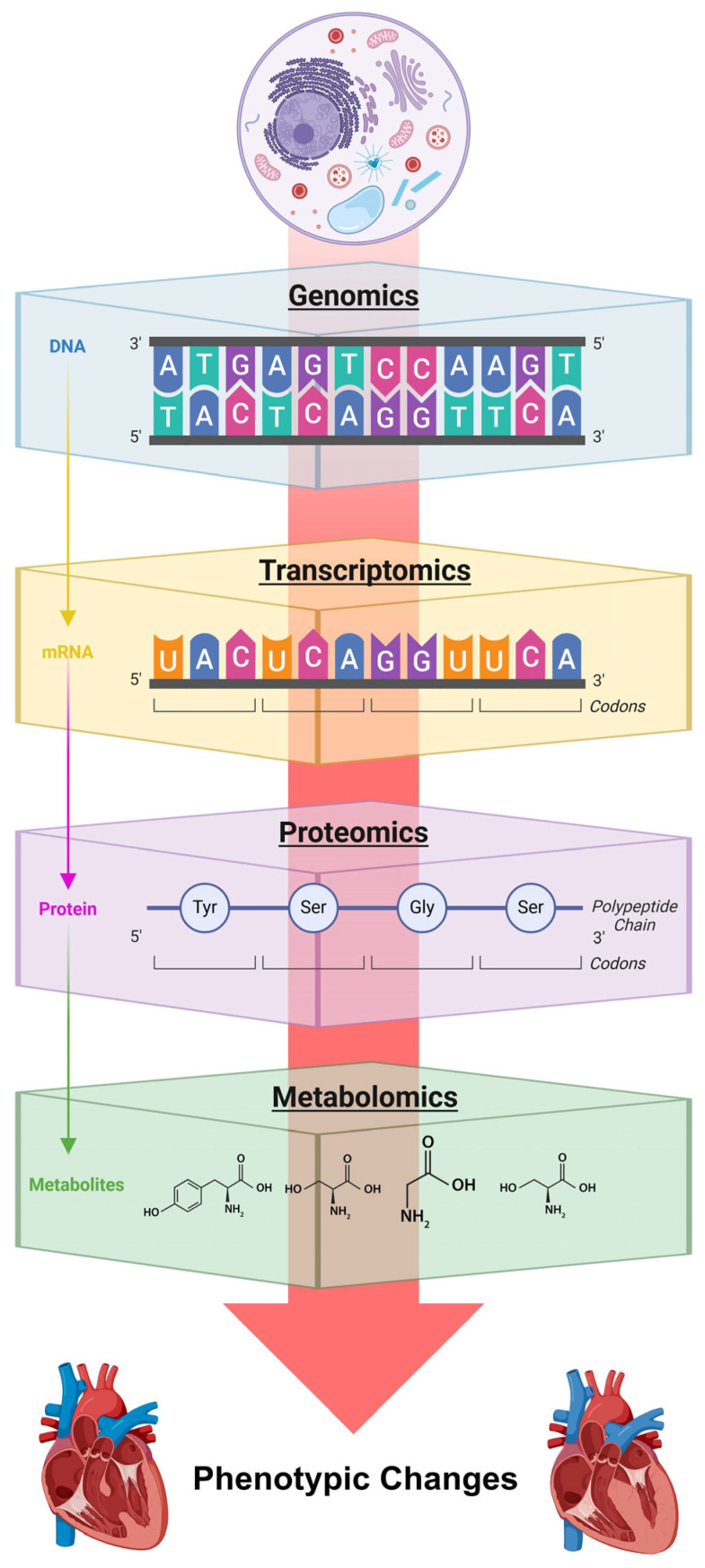
Basic concepts of the relationship between the genome, transcriptome, proteome, and metabolome. Together with other elements (such as the epigenome), these factors shape phenotypic changes, such as hypertrophic cardiomyopathy and dilated cardiomyopathy.

Recent advances in omics technologies (encompassing genomics, transcriptomics, proteomics, and metabolomics) have revolutionized HF research by providing comprehensive insights into its molecular mechanisms across multiple biological layers [37]. These tools have shifted the field from broad pathophysiological models to detailed molecular profiles, enabling the development of precise diagnostic tools and therapeutic strategies that pave the way for personalized medicine. This section explores how omics approaches illuminate HF’s complexity and their potential to transform clinical practice, summarized in Figure 3.

### 4.1. Genomic Foundations and Environmental Interactions

Genomic studies have revealed that HF susceptibility extends beyond single nucleotide polymorphisms to include structural variants, such as deletions, duplications, and inversions, particularly in inherited conditions like hypertrophic cardiomyopathy (HCM) and dilated cardiomyopathy (DCM) [38,39]. Rare genetic mutations in genes like MYH7 and TTN drive disease progression, underscoring the genetic underpinnings of these cardiomyopathies [39]. Environmental factors, including diet, lifestyle, and toxin exposure, interact with genetic predispositions, adding complexity to HF development [38]. Understanding these gene–environment dynamics has improved risk stratification, enabling family screening protocols and targeted preventive strategies, though predicting individual disease trajectories remains a significant challenge [40]. Some of the main genes involved in heart failure phenotypes are shown in Table 1.

MYH7 encodes β-myosin heavy chain (β-MHC), essential for myocardial contraction. Mutations in MYH7, mainly missense, account for up to 40% of HCM cases [57]. These alterations affect mitochondrial function and energy efficiency, especially in the ATP-binding head region [41]. G256E is a notable mutation causing conformational changes in the S1 head domain, linked to early-onset hypertrophy and increased arrhythmic risk [58,59]. MYH7 variants are also associated with restrictive/dilated cardiomyopathy and Ebstein’s anomaly. Despite a worse prognosis than MYBPC3-related HCM, genetic markers are not included in current risk models [41].

Emerging therapies include CRISPR base editing to correct variants like p.R403Q and RNA-based approaches (ASOs, RNAi) targeting mutant transcripts [60].

MYBPC3 encodes cardiac myosin-binding protein C (cMyBP-C), regulating sarcomere function. It is the most commonly mutated gene in HCM, representing ~44% of pathogenic variants [42]. About 75% of these are truncating mutations, leading to haploinsufficiency and, in biallelic cases, early-onset cardiomyopathy with poor prognosis.

No specific therapies are yet approved, but gene replacement using wild-type MYBPC3 cDNA is under investigation [61]. 

TTN encodes titin, the largest known sarcomeric protein, spanning from the Z-disc to the M-line. It provides structural integrity and functions as a molecular spring [43]. TTN truncating variants (TTNtv) are found in ~25% of familial and ~20% of sporadic DCM cases [62]. The pathogenicity depends on exon usage in cardiac isoforms; a PSI (percent spliced-in) > 15% correlates with phenotype penetrance. These variants increase the risk for heart failure and arrhythmias, and can be seen in restrictive or arrhythmogenic phenotypes [63]. Experimental therapies under evaluation include CRISPR-Cas9 editing, iPSC modeling, and gene overexpression via AAV vectors [64,65].

DES encodes desmin, a 53 kDa intermediate filament critical for sarcomere stabilization and linkage of Z-discs to cellular structures [66]. Mutations such as R406W and p.Y122H disrupt desmin filament organization, leading to cytoplasmic aggregation and impaired architecture [67]. These variants are associated with RCM but may also manifest as dilated or hypertrophic phenotypes [68]. Patients have high arrhythmic risk and a mortality rate of up to 50% within two years post-diagnosis [69]. Under investigation treatments include CRISPR-Cas9 and RNAi-based approaches [70].

Desmoplakin (DSP) is a key desmosomal component that links plaque proteins within intercalated discs, contributing to mechanical and electrical coupling [71]. The prevalent DSP isoform in the myocardium is DSP-1 [72]. Mutations like c.6154C>T (p.Gln2052Ter) lead to myocardial inflammation and fibrosis, significantly increasing arrhythmic risk [73]. Sudden cardiac death may be the first symptom in up to 50% of patients, prompting early ICD implantation. Though no targeted therapies exist, gene replacement efforts using AAV vectors (e.g., for PKP2 and PLN) show promise in preclinical models [74].

### 4.2. Advances in Transcriptomics and Regulatory RNA Networks

Transcriptomics has uncovered intricate regulatory mechanisms involving long non-coding RNAs (lncRNAs) and circular RNAs (circRNAs), which act as scaffolds for protein complexes and modulate alternative splicing in the failing heart [75]. The advent of spatial transcriptomics has further advanced the field by allowing researchers to map gene expression heterogeneity at cellular and tissue levels, revealing distinct transcriptional signatures across cardiac regions [76]. For instance, studies have identified upregulated fibrotic genes in peri-infarct zones following myocardial infarction (MI), providing insights into regional dysfunction in HF [77]. These RNA networks represent promising therapeutic targets, with early preclinical efforts exploring RNA-based interventions to modulate cardiac remodeling and restore function [78].

### 4.3. Proteomic Insights and Post-Translational Modifications

Quantitative and interaction-based proteomics have expanded our understanding of HF by identifying critical protein networks involved in contractile dysfunction and signaling cascades [79]. Post-translational modifications, such as acetylation and methylation, play a pivotal role in regulating protein function, influencing cardiac remodeling, energy metabolism, and cellular signaling [80]. Advanced proteomic techniques have uncovered novel interactions, such as acetylation-driven changes in mitochondrial proteins that contribute to energy deficits in HF, and have elucidated the mechanisms of existing therapies, such as beta-blockers, which modulate these pathways [81]. These findings position proteomics as a powerful tool for identifying therapeutic targets and biomarkers, although translating these insights into clinical applications requires further validation to ensure reproducibility and specificity [79].

### 4.4. Metabolomic Alterations and Cellular Energetics

Metabolomics has provided detailed insights into perturbations in lipid metabolism, amino acid processing, and nucleotide pathways, reflecting altered cellular energetics, protein turnover, and cell death in HF [82,83]. These metabolic shifts underscore the energy crisis in the failing heart, with reduced ATP production linked to mitochondrial dysfunction [84]. For example, studies have identified elevated branched-chain amino acid levels in HFrEF, correlating with disease severity, and altered lipid profiles as early markers of cardiac stress [85]. Mapping these metabolic changes has revealed potential targets, such as ketone metabolism, to improve energy efficiency, alongside biomarkers for early detection and risk stratification [86].

The integration of multi-omic datasets using AI and deep learning has facilitated the identification of precise biomarkers for HF diagnosis and prognosis, such as circulating microRNAs and proteomic signatures predictive of acute decompensation [87]. However, significant challenges remain, including the need to validate these biomarkers across diverse patient cohorts, interpret complex datasets, and address the high costs and limited accessibility of omics technologies. Ethical considerations, particularly regarding genomic data privacy and informed consent, pose additional hurdles, necessitating robust frameworks to ensure equitable application [87]. The future of omics in HF depends on overcoming these barriers, with the potential to deliver personalized treatments by combining genomic risk scores with metabolic and proteomic profiles, paving the way for a new era of precision cardiology [88].

## 5. Advanced Molecular Therapies

Advanced molecular therapies aim to regenerate myocardium or correct underlying genetic defects in HF, leveraging stem cell technologies and genetic modification techniques. These approaches hold transformative potential but are still evolving, with significant hurdles to overcome before widespread clinical adoption. 

### 5.1. Stem Cell Therapy in Heart Failure

Stem cell therapy seeks to repair damaged myocardium or stimulate endogenous repair mechanisms using mesenchymal stem cells (MSCs), induced pluripotent stem cells (iPSCs), and cardiac progenitor cells (CPCs) [89]. MSCs, derived from bone marrow or adipose tissue, exert therapeutic effects primarily through paracrine mechanisms, releasing cytokines such as vascular endothelial growth factor (VEGF), hepatocyte growth factor (HGF), and interleukin-10 (IL-10) to promote angiogenesis, reduce inflammation, and attenuate fibrosis [89]. A 2022 meta-analysis of 34 clinical trials in post-MI HF patients reported a modest improvement in left ventricular ejection fraction (LVEF) of 3.8% at 6 months, although 12-month mortality remained unchanged [90]. Lineage-tracing studies confirm that differentiation into functional cardiomyocytes is minimal (<1%), emphasizing the dominance of paracrine effects in MSC therapy [91].

### 5.2. Genetic Modification in Heart Failure

Genetic modification strategies aim to correct pathogenic mutations or enhance myocardial repair through targeted genome editing (e.g., CRISPR/Cas9, zinc finger nucleases (ZFNs), transcription activator-like effector nucleases (TALENs), and meganucleases) and non-integrative gene delivery (e.g., viral vectors, mRNA, and transposons) [92]. CRISPR/Cas9, valued for its simplicity and precision, has shown remarkable promise in preclinical models. For instance, it corrected an MYH7 mutation in hypertrophic cardiomyopathy (HCM) patient-derived iPSCs, restoring 90% of cardiomyocyte contractility in vitro, as measured by calcium transients [92]. In a murine model of laminopathy-associated dilated cardiomyopathy (DCM), adeno-associated virus serotype 9 (AAV9)-delivered CRISPR reduced fibrosis by 45% and improved LVEF by 14% at 8 weeks [93]. Post-MI, silencing transforming growth factor-beta 1 (TGF-β1) with CRISPR decreased scar formation by 30% and enhanced LVEF by 10% at 6 weeks in mice [94]. However, clinical translation is hampered by off-target effects (4% in 2023 porcine trials) and low delivery efficiency (15–25% of cardiomyocytes transduced), prompting an evaluation of advanced techniques like base editing and prime editing, which achieved 95% precision in 2024 murine models [95,96].

ZFNs, an earlier genome-editing tool, corrected a TTN mutation in iPSC-derived cardiomyocytes, improving contractility by 70% in vitro, while silencing connective tissue growth factor (CTGF) in ischemic HF models reduced fibrosis by 20% [97,98]. However, their labor-intensive design limits flexibility compared to CRISPR [99]. TALENs have been used to overexpress VEGF-A in post-MI murine models, increasing capillary density by 35% and LVEF by 8% at 8 weeks, while transposons like PiggyBac promote cardiomyocyte proliferation, offering alternative approaches [68,100]. AAV9 vectors delivering VEGF-A in porcine HF models boosted capillary density by 40% and LVEF by 11% at 12 weeks, but their limited cargo capacity (4.7 kb) and pre-existing immunity in 50% of humans pose significant barriers [101,102]. Synthetic mRNA delivered via lipid nanoparticles increased capillary density by 45% and LVEF by 11% at 4 weeks in post-MI mice, but storage challenges and high costs remain obstacles [103,104].

Synergistic approaches combining stem cells with genetic editing, such as CRISPR-edited iPSCs overexpressing GATA4 and TBX5 (92% differentiation efficiency) or TALEN-modified MSCs with HGF (35% less apoptosis and 40% more angiogenesis), enhance regenerative potential [105,106]. Advances in bioengineered scaffolds and safer delivery systems are addressing scalability and regulatory hurdles, with phase II/III trials anticipated by 2025. These strategies, while promising, must overcome combined limitations, such as cell retention and off-target effects, to achieve clinical success.

### 5.3. Other Therapies

Adenovirus-based gene therapy has shown promise in HF treatment by targeting molecular pathways critical to cardiac function. Intracoronary delivery of adenovirus encoding adenylyl cyclase type 6 (Ad5.hAC6) has been tested in clinical trials, demonstrating improved left ventricular function and remodeling in HF patients through enhanced cyclic AMP production. Similarly, adenovirus-mediated delivery of βARKct, a peptide inhibiting GRK2-mediated β-adrenergic receptor desensitization, has reversed ventricular dysfunction in preclinical models, offering a potential strategy to restore cardiac contractility [107]. 

In contrast, PRINT (Precise RNA-mediated Insertion of Transgenes for Medicine) and PASTE (Programmable Addition via Site-specific TransEditing) represent cutting-edge RNA-guided gene-editing technologies. These approaches leverage CRISPR-based systems to insert or edit therapeutic genes with high precision, potentially correcting genetic defects underlying cardiomyopathies that lead to HF. While still in early development, their ability to target specific genomic loci could revolutionize personalized HF treatments [108]. 

BASE (Base Editing for Adenine and Cytosine) therapy, another CRISPR-derived technique, enables precise single-nucleotide edits without double-strand breaks, offering a safer alternative for correcting point mutations associated with HF susceptibility. Though preclinical studies are promising, challenges such as off-target effects and delivery efficiency remain [109]. 

These therapies, combined with omics-driven insights, hold immense potential for precise, mechanism-based HF interventions, though further validation is needed to ensure safety and efficacy in clinical settings.

## 6. Personalized Medicine in Heart Failure

**Figure 4:**
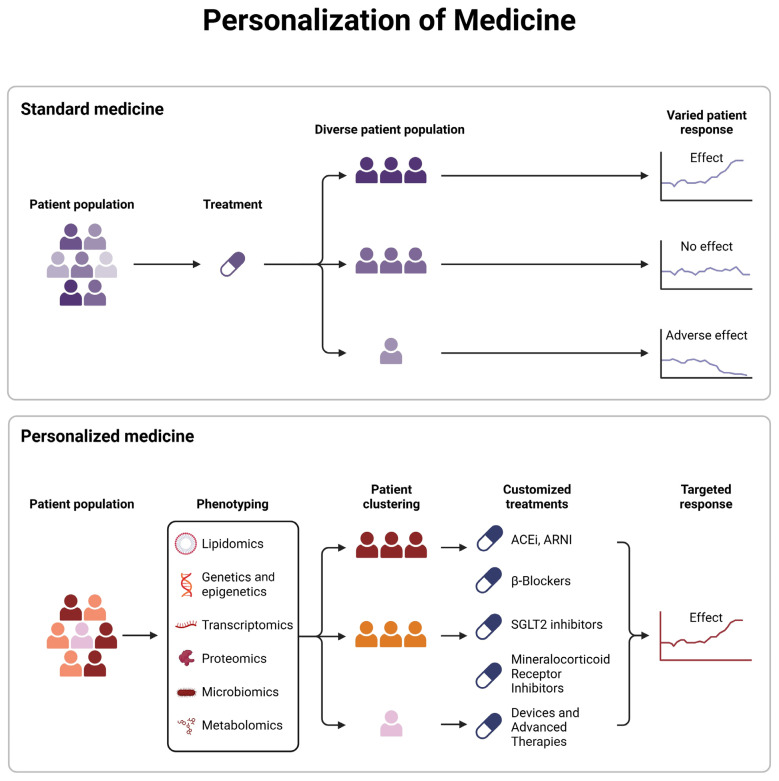
The key difference between personalized and standard medicine lies in identifying similar characteristics in phenotypic aspects (lipidome, genome, microbiome, metabolome, etc.), which influence specific responses and advantages. This approach adjusts treatments not only based on clinical or demographic characteristics but also on cellular and molecular factors.

Personalized medicine in HF leverages molecular profiling through genomics, transcriptomics, epigenomics, and proteomics to tailor diagnostic and therapeutic strategies to individual patient profiles, addressing variability in disease mechanisms such as oxidative stress and mitochondrial function [110]. By integrating these data, clinicians can optimize treatment responses, moving beyond one-size-fits-all approaches to deliver precision care that accounts for genetic, environmental, and phenotypic differences (Figure 4).

Personalized medicine harnesses molecular profiling to tailor therapies to individual patient profiles, addressing the diverse pathophysiology of HFrEF and HFpEF [110,111]. Established treatments, such as beta-blockers and ARNIs, target endothelial dysfunction and oxidative stress, achieving significant mortality reductions in HFrEF (up to 64%) by modulating mitochondrial function and the natriuretic pathway [112,113]. In specific contexts, such as diabetic cardiomyopathy, metformin enhances autophagy via AMP-activated protein kinase activation, while statins stabilize endothelial nitric oxide synthase to reduce inflammation [110,111]. These therapies highlight the potential of molecularly guided interventions, yet their variable efficacy across HF phenotypes underscores the need for precision approaches that integrate clinical and molecular insights. Such strategies, particularly for HFpEF, will be explored in the following section to optimize outcomes for this challenging condition.

## 7. HFpEF: Converging Clinical and Molecular Perspectives

HFpEF, marked by impaired diastolic function and exercise intolerance, arises from a complex interplay of mitochondrial dysfunction and reactive oxygen species (ROS), driven by comorbidities such as obesity, type 2 diabetes, and hypertension [110,114,115]. In contrast to HFrEF, HFpEF lacks therapies that reliably enhance survival, underscoring the urgent need for innovative strategies that address its molecular underpinnings while tackling clinical challenges. A summary of the main pharmacological interventions is shown in Table 2.

### 7.1. Pharmacological Interventions

a. Mitochondrial dysfunction and HFpEF: Mitochondrial dysfunction is a central and multifaceted contributor to the pathophysiology of heart failure, with reactive oxygen species (ROS) and oxidative stress emerging as critical drivers of disease progression. Mitochondria, being the primary source of ROS in cardiomyocytes, generate these molecules during oxidative phosphorylation. Under pathological conditions (such as disrupted electron transport and impaired membrane potential) ROS production is amplified, particularly at complexes I and III, initiating a self-perpetuating cycle of ROS-induced ROS release [116]. This oxidative overload damages mitochondrial DNA, proteins, and lipids, leading to membrane depolarization, mPTP opening, and cell death through apoptosis or necrosis. 

A key regulator in resisting these detrimental effects is PGC-1α (peroxisome proliferator-activated receptor gamma coactivator-1 alpha), a transcriptional coactivator that orchestrates mitochondrial biogenesis, oxidative metabolism, and antioxidant defenses [117]. Upon energetic stress, PGC-1α activates nuclear respiratory factors (NRF1 and NRF2) and mitochondrial transcription factor A (TFAM), boosting mitochondrial replication and respiratory capacity. It also promotes the expression of endogenous antioxidants, helping to buffer ROS accumulation. However, in chronic heart failure, this adaptive response becomes blunted (PGC-1α signaling is frequently suppressed, diminishing the cell’s ability to recover mitochondrial function and maintain redox balance) [116]. The failure of this compensatory pathway contributes to a downward spiral of mitochondrial deterioration, bioenergetic collapse, and heightened inflammatory signaling via DAMPs such as oxidized mtDNA. 

Promising therapeutic strategies aim to restore or enhance PGC-1α activity. For example, structured exercise programs can upregulate PGC-1α expression, promoting mitochondrial renewal and antioxidant defenses, while dietary supplements like nicotinamide riboside (NR) boost NAD+ availability, supporting PGC-1α-dependent pathways [117]. Despite encouraging preclinical results, clinical translation remains in early stages, though these interventions represent a promising direction for targeting the energetic and oxidative roots of heart failure.

On the other hand, elamipretide (SS-31), a tetrapeptide that stabilizes cardiolipin in the mitochondrial inner membrane, reduces ROS, and enhances respiratory efficiency. In the 2023 PROGRESS-HF phase II trial, elamipretide (4 mg/day, subcutaneous) improved diastolic function, reducing the E/e’ ratio by 15%, and increased exercise capacity (+32 m in the 6 min walk test) at 12 weeks, with a favorable safety profile [114]. Larger trials are needed to confirm its impact on clinical endpoints like hospitalization or mortality.

b. Sodium-glucose cotransporter 2 inhibitors: SGLT2is, such as empagliflozin, enhance myocardial energy efficiency by promoting ketone body utilization and reducing ROS through inhibition of the sodium-hydrogen exchanger (NHE1) and upregulation of antioxidant enzymes. The EMPEROR-Preserved trial demonstrated that empagliflozin (10 mg/day) reduced heart failure hospitalizations by 17% in HFpEF patients, with benefits linked to decreased inflammation and improved mitochondrial function [115]. A 2024 murine study reported a 12% increase in mitochondrial ATP production with empagliflozin, underscoring its bioenergetic effects [118].

SGLT2is promote natriuresis by inhibiting sodium reabsorption in the proximal tubule through blockade of the sodium–hydrogen exchanger 3 (NHE3), reducing extracellular volume and myocardial edema [119]. This decrease in preload lowers left ventricular end-diastolic pressure, alleviating myocardial stiffness and improving diastolic function, key hallmarks of HFpEF. Cardiac magnetic resonance studies have demonstrated significant reductions in left ventricular mass in HFpEF patients following SGLT2i initiation, reflecting attenuated myocardial remodeling and improved cardiac mechanics [120]. By modulating neurohumoral responses, SGLT2i also mitigates sympathetic overactivation, further supporting cardiovascular homeostasis and reducing the neurohormonal burden that exacerbates HF progression.

SGLT2i-induced glucosuria leads to caloric loss, inducing a state of metabolic restriction akin to fasting, particularly in energy-demanding tissues like the heart, skeletal muscle, and kidneys [121]. This metabolic shift triggers the activation of sirtuins (Sirt1, Sirt3, and Sirt6), NAD+-dependent deacetylases that regulate autophagy, mitochondrial function, and cellular stress responses [121,122]. Autophagy, the cellular process of recycling dysfunctional proteins and organelles, is impaired in HFpEF, contributing to oxidative stress and inflammation that drive disease progression [122]. Sirt1 promotes autophagy by deacetylating autophagy-related genes (Atg5, Atg7, and Atg8) and the transcription factor forkhead box O1 (FoxO1), enhancing the expression of autophagic regulators [123]. This process reduces oxidative stress, mitigates inflammation, and protects cardiomyocytes from metabolic imbalance, which otherwise leads to contractile dysfunction, microvascular rarefaction, fibrosis, and extracellular matrix remodeling [124]. By restoring NAD+ levels, which are often depleted in HFpEF, SGLT2is reorganize cardiomyocyte metabolic cascades, offering a novel cardioprotective mechanism that complements their natriuretic effects.

Emerging evidence suggests that SGLT2is may induce mild, persistent hyperketonemia, potentially via the Sirt1/PGC-1α pathway, where β-hydroxybutyrate is preferentially oxidized over fatty acids, enhancing myocardial energy efficiency [121,125]. Ketone bodies also exhibit anti-inflammatory properties by inhibiting the NLRP3 inflammasome and stimulating autophagy, potentially reducing cytokine-induced mitochondrial damage and cardiac fibrosis in HFpEF [125,126]. However, the contribution of ketosis to HFpEF benefits remains controversial, as clinical evidence is limited, and the precise mechanisms underlying ketone-mediated cardioprotection are still under investigation. Nevertheless, this metabolic shift may improve energy utilization, offering a complementary mechanism to natriuresis and sirtuin activation, and warrants further exploration to clarify its therapeutic potential [126].

c. Glucagon-like peptide-1 receptor agonists (GLP-1RAs): GLP-1RAs, such as semaglutide, address obesity-driven oxidative stress in HFpEF by reducing visceral fat and systemic inflammation. In the 2023 STEP-HFpEF trial, semaglutide (2.4 mg/week) improved exercise capacity (+21 m in the 6 min walk test) and reduced NT-proBNP levels by 15% in obese HFpEF patients, with effects attributed to decreased ROS and enhanced mitochondrial biogenesis via AMPK activation [127]. These findings are particularly relevant for HFpEF patients with metabolic syndrome.

d. Nitroxyl donors: Nitroxyl (HNO) donors, such as BMS-986231, enhance mitochondrial function and calcium cycling without adrenergic stimulation, reducing oxidative stress. In a 2024 phase II trial, BMS-986231 (7 µg/kg/min infusion) improved myocardial relaxation (reduced relaxation time by 8%) and lowered ROS markers by 18% at 8 weeks in HFpEF patients, without significant hypotensive effects [128]. Phase III trials are underway to evaluate long-term outcomes.

e. Mineralocorticoid receptor antagonists: Finerenone, a non-steroidal MRA, curbs aldosterone-mediated ROS production, reducing fibrosis and cellular stress. The 2024 FINEARTS-HF trial reported that finerenone (10–20 mg/day) lowered cardiovascular events (death or hospitalization) by 16% in HFpEF patients, particularly those with diabetes [129]. Mechanistic studies indicate finerenone boosts mitochondrial complex I activity, increasing ATP production by 10% in HFpEF models [130]. Other mineralocorticoid receptor antagonists (spironolactone) mitigate oxidative stress in ischemic HF, increasing endothelial progenitor cells (VEGFR2+/CD34+) [110,112,118].

f. Beta-blockers: Non-selective beta-blockers such as carvedilol (acting on β2 and α1 receptors) reduce mitochondrial oxygen consumption and ROS, achieving a 64% mortality reduction in 1996, unlike metoprolol, while improving L-arginine, L-citrulline, and vascular cell adhesion molecule-1 (VCAM-1) levels [110,121].

g. Others: In diabetic cardiomyopathy, metformin enhances autophagy via AMPK activation, increasing LC3-II and mitochondrial respiration [110]. Statins offer anti-inflammatory and antioxidant effects, stabilizing eNOS mRNA, reducing TNF-α, and promoting CD34+ cell activation for neovascularization and LVEF improvement [111]. Pharmacological inhibition of dynamin-related protein-1 (DRP-1) reduces cell death post-ischemia–reperfusion, while antioxidants (e.g., allopurinol) and Szeto-Schiller peptides (e.g., SS-31 in PROGRESS-HF) show limited structural benefits [110,113].

### 7.2. Iron Deficiency Correction

Iron deficiency, affecting up to 50% of HFpEF patients, significantly impairs mitochondrial oxidative phosphorylation and exacerbates exercise intolerance, even in the absence of anemia [131]. This condition disrupts iron availability for heme synthesis and mitochondrial enzyme function, contributing to energy deficits and cellular stress that compound HFpEF pathophysiology. Intravenous iron supplementation, such as ferric carboxymaltose, addresses this deficit by restoring iron stores, thereby enhancing mitochondrial ATP production and improving functional capacity. The CONFIRM-HF trial and subsequent HFpEF subanalyses conducted in 2023 demonstrated that ferric carboxymaltose, dosed according to iron deficit, improved 6 min walk test distance by 28 m and quality of life scores at 6 months, with benefits sustained at 12 months [131]. These effects, driven by enhanced mitochondrial bioenergetics, complement the actions of SGLT2i, which similarly target energy deficiency and oxidative stress. Integrating routine iron screening and correction into HFpEF management, particularly for patients with reduced functional capacity, offers a practical and synergistic approach to optimize outcomes alongside SGLT2i therapy. By addressing these overlapping pathways, clinicians can enhance personalized care, tailoring interventions to the metabolic and functional needs of HFpEF patients.

### 7.3. Non-Pharmacological Interventions: Exercise Training

Structured exercise, combining aerobic and resistance training, promotes mitochondrial biogenesis and upregulates antioxidant enzymes, such as superoxide dismutase. A 2024 randomized controlled trial in HFpEF patients demonstrated that 12 weeks of supervised aerobic training (3 sessions/week at 60% VO_2_ peak) increased mitochondrial DNA content by 15% and improved VO_2_ by 2.5 mL/kg/min, alongside reduced oxidative stress markers [132]. Exercise also enhances nitric oxide bioavailability, supporting endothelial function and diastolic relaxation [121]. However, adherence remains a significant challenge, particularly among elderly patients, necessitating strategies to improve compliance.

While these interventions offer exciting prospects for HFpEF, several challenges persist. The heterogeneity of HFpEF phenotypes, driven by varying comorbidities, complicates treatment efficacy, as responses differ across patient subgroups. For instance, SGLT2i and GLP-1RAs are most effective in obese or diabetic patients, while mitochondrial-targeted therapies like elamipretide may benefit those with pronounced bioenergetic deficits. The high costs of novel agents and the lack of robust evidence for mortality benefits further hinder widespread adoption. Technical challenges in clinical trials, such as defining appropriate endpoints for diastolic function or mitochondrial health, also require careful consideration.

Future research should prioritize personalized approaches, leveraging multi-omics (e.g., metabolomics and proteomics) to identify patients likely to respond to specific therapies [87]. Combining pharmacological agents, such as SGLT2i with GLP-1RAs or elamipretide, could synergistically address mitochondrial dysfunction and oxidative stress, meriting investigation in phase III trials. Additionally, scalable strategies to improve exercise adherence, such as telemonitored programs, could enhance the impact of non-pharmacological interventions. By 2027, ongoing trials for elamipretide, nitroxyl donors, and combination therapies may clarify their role in HFpEF, potentially shifting management toward targeted, bioenergetic-focused care that addresses the unique needs of this patient population.

## 8. Conclusions, Challenges, and Future Directions

HF integrates complex mechanisms, from RAAS overactivation and neurohormonal imbalances to emerging pathways like the gut–heart axis and mitochondrial dysfunction. RAAS inhibitors and ARNIs have transformed HFrEF management, while novel therapies, including SGLT2i, GLP-1RAs, and elamipretide, address unmet needs in HFpEF, enhancing functional capacity. Stem cell therapies improve LVEF but face retention challenges, and CRISPR/Cas9 shows preclinical promise despite off-target risks. TMAO-targeted interventions, such as dietary and probiotic strategies, offer potential but require validation. Omics technologies enable personalized biomarkers, yet high costs, patient adherence, HFpEF’s phenotypic heterogeneity, and validation challenges hinder progress. By 2030, global collaboration could drive regenerative and precision-based HF care, integrating AI-driven omics to tailor therapies and developing cost-effective diagnostics.

SGLT2i and MRA can be readily prescribed for HFpEF patients with comorbidities like diabetes, while dietary counseling to reduce TMAO is feasible in primary care. However, high costs of novel agents, variable patient adherence, and HFpEF’s heterogeneity limit widespread adoption, necessitating omics-based stratification tools to personalize treatments. Overcoming these barriers through rigorous trials and AI-supported risk prediction will enhance equitable access and optimize outcomes in this complex disease.

## Figures and Tables

**Table 1:** Genetic involvement in heart failure phenotypes.

Phenotype	Involved Genes	Mutation	Gene Target	Cellular/Cardiac Effect	References
HCM	*MYH7*	Missense mutation (G256E)	β-Myosin heavy chain	ATPase activity, force generation	[41]
	*MYBPC3*	Mutations are truncating, resulting in the absence of protein.	Myosin-binding protein C	Cardiac contraction	[41,42]
	*TNNT2*	Missense mutation/deletion	Cardiac troponin T	Regulator of actomyosin interaction	[41]
	*TNNI3*	Missense mutation (p.Glu125Gly)	Cardiac troponin I	Inhibitor of actomyosin interaction	[41]
	*TPM1*	Missense mutation (c.842T>C, p.Met281Thr)	α-tropomyosin	Places the troponin complex on cardiac actin	[41,42]
	*ACTC1*	Ala21Val mutation	Cardiac α-actin	Actomyosin interaction	[41]
	*MYL2*	Intronic mutation (IVS6-1)	Regulatory myosin light chain	Myosin heavy chain 7-binding protein	[41]
	*MYL3*	Missense mutation (Ala57Gly)	Essential myosin light chain	Myosin heavy chain 7-binding protein	[41]
	*CSRP3*	Missense mutations, nonsense mutations	Cysteine- and glycine-rich protein 3	Muscle LIM protein (MLP), a Z disk protein	[41,42]
DCM	*RBM20*	Missense mutations	RNA-binding motif protein20	Aggressive DCM, malignant ventricular arrhythmias	[43]
	*MYH7*	Missense mutation	B-myosin heavy chain	ATPase activity, force generation	[43]
	*DSG2*	Missense mutation (V55M and V919G)	Desmoglein-2	ARVC, ventricular arrhythmia, sudden cardiac death (woolly hair, keratoderma)	[43]
	*TTN*	Missense mutation/deletion	Titin	Structural and functional sarcomere support	[43]
	*DES*	Missense mutation Ile451Met	Desmin	ARVC, skeletal myopathy	[43]
	*SCN5A*	Missense mutation	Voltage-gated sodium channel	Skeletal myopathy, Long QT syndrome	[43]
	*LMNA*	LMNA-C.185G>C (p.Arg62Pro)	Lamin A/C	Cardiac conduction abnormalities, arrhythmic abnormalities	[43]
	*DSP*	c.405_422+39del	Desmoplakin	ARVC, right bundle branch block, right and left ventricular arrhythmias, DCM	[43]
ACM	*DSP*	c.6154C>T p.Gln2052Ter	Desmoplakin	ARVC, right bundle branch block, right and left ventricular arrhythmias, DCM	[44,45]
	*DSG2*	p.Arg119Ter	Desmoglein-2 protein	Disrupt the structural integrity of the heart muscle, leading to fibrofatty replacement	[46,47]
	*DSC2*	Antisense	Desmocollin-2 protein	Disruption of desmosome	[48,49]
	*PLN*	Mutation Arg14del	Phospholamban	Disrupt calcium handling in heart muscle cells, leading to a range of cardiac issues, including ventricular arrhythmias	[50,51]
	*TMEM43*	Missense mutation p.S358L	LUMA protein	ARVC type 5	[52,53]
RCM	*DES*	p.Y122H R406W	Desmin protein	Severe filament assembly defect, also associated with high risk of ventricular arrhythmias	[54,55]
	*FLNC*	Missense NM_001458.4:c.6902C>T, p.Pro2301Leu	Filamin C	Abnormal protein aggregation and disorganized sarcomeres	[55]
	*BAG3*	BAG3-Pro209Leu	BAG3 protein	Myofibrillar myopathy (MFM), causing progressive muscle weakness, respiratory issues, and neuropathy	[56]

HCM: hypertrophic cardiomyopathy, DCM: dilatated cardiomyopathy, ACM: arrhythmogenic cardiomyopathy, and RCM: restrictive cardiomyopathy.

**Table 2:** Summary of HFpEF therapies and their pathways in heart failure.

Therapy	Pathway
Mitochondrial-Targeted Antioxidants (Elamipretide)	Cardiolipin stabilization in the mitochondrial inner membrane. Reduces ROS, enhances respiratory efficiency.
Sodium/Glucose Transporter 2 Inhibitors (SGLT2i) (Empagliflozin, Dapagliflozin)	Myocardial energy efficiency. Natriuresis and neurohumoral modulation. Autophagy and sirtuin activation. Ketogenic metabolism.
Glucagon-like Peptide-1 Receptor Agonists (GLP-1RAs) (Liraglutide, Semaglutide)	Obesity-driven oxidative stress. Visceral fat reduction.
Nitroxyl Donors (BMS-986231)	Enhance mitochondrial function and calcium cycling without adrenergic stimulation.
Mineralocorticoid Receptor Antagonists (Spironolactone, Eplerenone, Finerenone)	Reduction in aldosterone-mediated ROS production. Finerenone boosts mitochondrial complex I activity. Spironolactone increases endothelial progenitor cells (VEGFR2+/CD34+).
Beta-Blockers (Carvedilol, Metoprolol, Bisoprolol)	Carvedilol reduces mitochondrial oxygen consumption and ROS. Metoprolol rises L-Arginine, L-citrulline, and VCAM-1.
Biguanides (Metformin)	Autophagy by AMPK activation, increasing LC3-II and mitochondrial respiration.
Statins (Rosuvastatine, Atorvastatine)	Stabilize eNOS mRNA, reduce TNF-A, and promote CD34+ activation.
Dynamin-related Protein-1 (DRP-1) Inhibitors (mdivi-1)	Changes in mitochondrial dynamics, autophagy, ATP production, immune response, and calcium homeostasis. Reduces cell death post ischemia-reperfusion.
Funny Current (If) Inhibitors (Ivabradine)	Inhibits HCN4 channels in the sinoatrial node, slows diastolic depolarization, and reduces heart rate without affecting contractility.
Soluble Guanylate Cyclase Stimulators (Vericiguat)	Stimulates soluble guanylate cyclase independently of NO. Increases cGMP and activates PKG, promoting vasodilation, anti-fibrotic, and cardioprotective effects.
Cardiac Myosin Activators (Omecamtiv mecarbil)	Activates cardiac myosin (S1 domain), stabilizes pre-power stroke state, prolongs systolic ejection time and increases contractility without raising intracellular Ca^2+^ or oxygen demand.

## Data Availability

Not applicable.
